# Multimorbidity healthcare expenditure in Belgium: a 4-year analysis (COMORB study)

**DOI:** 10.1186/s12961-024-01113-x

**Published:** 2024-03-22

**Authors:** Phuong Bich Tran, Georgios F. Nikolaidis, Emmanuel Abatih, Philippe Bos, Finaba Berete, Vanessa Gorasso, Johan Van der Heyden, Joseph Kazibwe, Ewan Morgan Tomeny, Guido Van Hal, Philippe Beutels, Josefien van Olmen

**Affiliations:** 1 Department of Family Medicine and Population Health, University of Antwerp, Antwerp, Belgium; 2 IQVIA Ltd, 37 North Wharf Road, London, W2 1AF UK; 3 Department of Applied Mathematics, Computer Sciences and Statistics, Ghent University, Ghent, Belgium; 4 Department of Sociology, University of Antwerp, Antwerp, Belgium; 5 Department of Epidemiology and public health, Brussels, Belgium; 6 Department of Clinical Sciences, Lund University, Malmö, Sweden; 7 Department of Clinical Sciences, Liverpool School of Tropical Medicine, Liverpool, UK; 8 Centre for Health Economics Research & Modelling Infectious Diseases (CHERMID), University of Antwerp, Antwerp, Belgium

**Keywords:** Cost analysis, Healthcare expenditure, Multimorbidity, Chronic diseases, Noncommunicable diseases, Disease interaction, Integrated care, Belgium

## Abstract

**Background:**

The complex management of health needs in multimorbid patients, alongside limited cost data, presents challenges in developing cost-effective patient-care pathways. We estimated the costs of managing 171 dyads and 969 triads in Belgium, taking into account the influence of morbidity interactions on costs.

**Methods:**

We followed a retrospective longitudinal study design, using the linked Belgian Health Interview Survey 2018 and the administrative claim database 2017–2020 hosted by the Intermutualistic Agency. We included people aged 15 and older, who had complete profiles (*N* = 9753). Applying a system costing perspective, the average annual direct cost per person per dyad/triad was presented in 2022 Euro and comprised mainly direct medical costs. We developed mixed models to analyse the impact of single chronic conditions, dyads and triads on healthcare costs, considering two-/three-way interactions within dyads/triads, key cost determinants and clustering at the household level.

**Results:**

People with multimorbidity constituted nearly half of the study population and their total healthcare cost constituted around three quarters of the healthcare cost of the study population. The most common dyad, arthropathies + dorsopathies, with a 14% prevalence rate, accounted for 11% of the total national health expenditure. The most frequent triad, arthropathies + dorsopathies + hypertension, with a 5% prevalence rate, contributed 5%. The average annual direct costs per person with dyad and triad were €3515 (95% CI 3093–3937) and €4592 (95% CI 3920–5264), respectively. Dyads and triads associated with cancer, diabetes, chronic fatigue, and genitourinary problems incurred the highest costs. In most cases, the cost associated with multimorbidity was lower or not substantially different from the combined cost of the same conditions observed in separate patients.

**Conclusion:**

Prevalent morbidity combinations, rather than high-cost ones, made a greater contribution to total national health expenditure. Our study contributes to the sparse evidence on this topic globally and in Europe, with the aim of improving cost-effective care for patients with diverse needs.

**Supplementary Information:**

The online version contains supplementary material available at 10.1186/s12961-024-01113-x.

## Background

A new challenge has arisen in global health. Traditionally, approaches to morbidity management have used classifications such as ‘communicable/noncommunicable’ and ‘chronic/acute’. Today, we are facing a more complex phenomenon as an increasing number of people across the globe live with multimorbidity [1]. Multimorbidity is often defined as the co-occurrence of two or more chronic conditions in the same person [3]. Comparing multimorbidity rates between studies is challenging as there are significant differences in the populations and the methodological choices made. However, the standardised prevalence rate of multimorbidity has been shown to rise significantly for all sexes and age groups between 2000 and 2015 in Belgium [4]. The increase in multimorbidity is frequently attributed to extended life expectancy and improvements in the management of chronic illnesses [5]. In Belgium, however, multimorbidity is also rising fast among younger age groups. For those under 50 years of age, the standardised prevalence rate of multimorbidity doubled between 2000 and 2015 [4].

While clinico-pathological research is ongoing, it is becoming more apparent how multimorbidity affects healthcare systems and societies as a whole [6, 7]. The management of health needs in these patients is creating challenges in the development of cost-effective patient-care pathways, posing heavy economic burdens on households, health systems and societies [2]. Moreover, the complexity of managing multiple conditions often requires a more intricate and coordinated approach from healthcare providers, increasing the demand for specialised and integrated care services [8]. This, in turn, places strains on healthcare systems, potentially leading to resource allocation dilemmas and access disparities [9]. The increased healthcare visits, medications and treatment burden – i.e., the actions and resources patients devote to their healthcare, including difficulty, time and resources dedicated to the healthcare tasks such as adhering to medications, dietary recommendations and self-monitoring – can result in escalated healthcare costs [10, 11]. Workforce productivity may decline, while care-giving demands intensify, impacting both the economy and social support networks [12].

Multimorbidity presents significant challenges for healthcare systems across the board, including high-income countries. In Belgium, a fragmented care system can exacerbate the issues of increased healthcare utilisation and costs, as well as difficulties in coordinating and managing care [4, 13]. Belgium’s healthcare system allows patients and providers considerable freedom of choice, with fee-for-service payments as the primary mode of remuneration [14]. Unlike gatekeeping systems, patients can consult any general practitioner or specialist [14]. While acute care is commendable, there is room to bolster primary care and improve care coordination to address the increasing challenges posed by a rising number of individuals with chronic illnesses [14].

A recent systematic review on the costs of multimorbidity highlighted how research on this topic is limited in both scope and number [2]. Previous studies have typically been limited to either ‘cost-per-disease-count’ or ‘cost-per-additional-disease’, and relying on data obtained from a single source. The research originated from a small pool of countries (predominantly the United States), with a striking lack of published studies on multimorbidity costs from Europe and low- and middle-income countries. Cost of multimorbidity studies focusing on specific morbidity combinations are often limited to a single or a few combinations centred around an index disease of concern. Only a limited number of studies have taken a comprehensive approach, analysing a broad array of morbidity combinations at the population level. These studies are from the United States, the United Kingdom and New Zealand [15–17].

Further research is needed to understand the costs of specific combinations of chronic conditions, as a basis for identifying and further exploring where and how costs can be averted/reduced while ensuring high-quality care. Dyads and triads – characterised by the coexistence of two and three conditions respectively – stand out as prominent subjects of multimorbidity research due to their prevalence within multimorbidity populations, making them crucial targets for investigation. Concurrently, direct costs – which entail expenses tied to healthcare resources, interventions or services – have garnered significant attention in cost analyses due to their tangible and immediate impact on healthcare budgets. The lack of understanding of the direct costs of morbidity combinations – or the interaction of individual conditions and its impact on cost – poses a challenge for policy-makers in developing new models of integrated patient-centred care and evaluating their cost-effectiveness. Through this study, we hope to deliver a holistic health system perspective of healthcare costs for people with multimorbidity and provide formative evidence to inform the re-organisation of healthcare delivery to support patients with multiple needs and promote the efficient use of healthcare resources.

This study aims to estimate the economic burden of multimorbidity in Belgium and to explore the interactions of coexisting chronic conditions in a person and how they influence healthcare costs.

Particularly, the research questions are:

1. How do the direct healthcare costs of multimorbidity vary across population subgroups in Belgium?
2. What is the average annual direct healthcare cost per person per single chronic condition, dyad and triad?
3. Across dyads, is the direct cost more, less or equal to the expected combined direct cost of single conditions? Across triads, is the direct cost more, less or equal to the expected combined direct cost of the three associated dyads?
4. Which dyads/triads have the most economic impact on the individual and population level?

## Methodology

### Design

This is a retrospective longitudinal study using data from two databases. The study was reported in line with the Reporting of studies Conducted using Observational Routinely-collected Data (RECORD) guidelines [18] and the Consolidated Health Economic Evaluation Reporting Standards (CHEERS) 2022 [19].

### Data sources

The first database is the 2018 Belgian Health Interview Survey (BHIS). The BHIS contains data on socio-demographic characteristics, health status, health behaviours, healthcare utilisation and quality of life as well as other health determinants from a sample of over 10,000 people from approximately 6000 households in Belgium [20]. The BHIS is a cross-sectional design survey, which uses the national register as its sample frame. It is conducted every 4–5 years to allow for comparison of population changes over time, and serves as a basis to inform health service organisation and policy. The BHIS aligns and follows the format of the European Health Interview Survey (EHIS). The sampling technique and implementation of the BHIS have been published elsewhere [21].

The second database is the Intermutualistic Agency (IMA) database, an administrative database that collects data on the individual-level usage and costs of healthcare from all public health insurance funds [22]. Every legal resident in Belgium is obliged to join one of the seven Belgian health insurance funds and is therefore included in the IMA database. Healthcare reimbursement data makes up the core of the database and data has been updated twice every year from 2002 onwards. There are three sub-databases: (1) A population database containing limited socio-demographic data of all insured persons; (2) A healthcare database containing healthcare service utilisation and cost data of ambulatory and hospital care; and (3) A pharmaceutical database containing prescriptions and the costs of prescribed and reimbursed drugs purchased through public pharmacies. The IMA data utilised in this study spanned across four years, from 2017 to 2020. Data from the BHIS and IMA databases were linked using the national register number, forming the integrated HISLINK database.

### Study participants

**Fig. 1:**
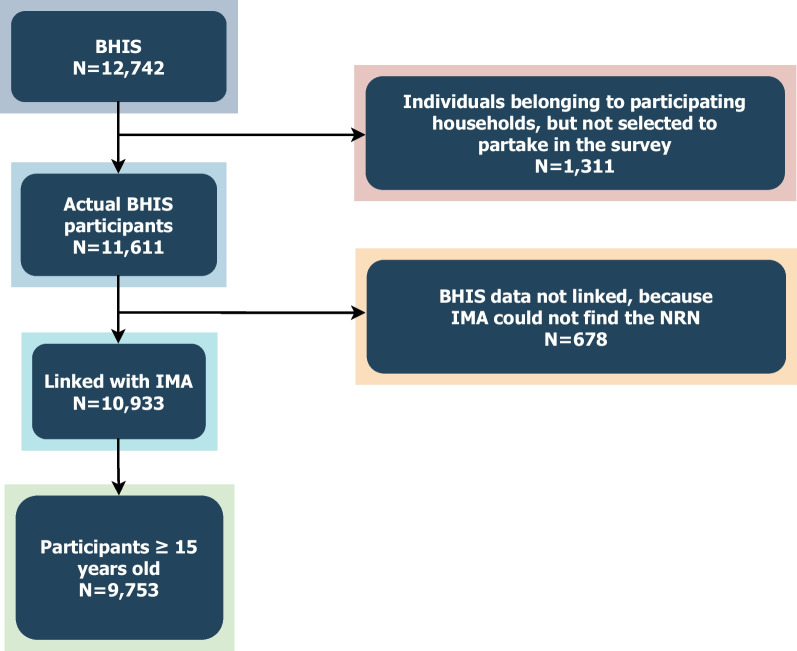
Sample selection

We included all community-living people in Belgium, aged 15 and older, who had complete profiles and successful data linkage, from the HISLINK database (*N* = 9753) (Fig. 1). We analysed participants’ sex (male/female), age group (10-year intervals), highest education level in the household (no diploma or primary education, lower secondary, higher secondary, higher education), annual household income in 2018 Euro (five quintiles), region (Flemish, Wallonia, Brussels) and number of chronic conditions (0, 1, 2, 3, 4+).

### Chronic conditions

The presence of chronic conditions was self-reported and was determined through the interviewee’s response to the question: “During the past 12 months, have you had any of the following diseases or conditions?”. Information on 38 chronic conditions was collected in the BHIS, which was subsequently regrouped to 25 chronic conditions or morbidity groups as many conditions had shared or similar pathophysiology (Table 1). The mapping was adapted from Van Wilder et al. [23], the International Statistical Classification of Diseases and Related Health Problems tenth Revision [24], and discussion among the team of authors.

**Table 1:**
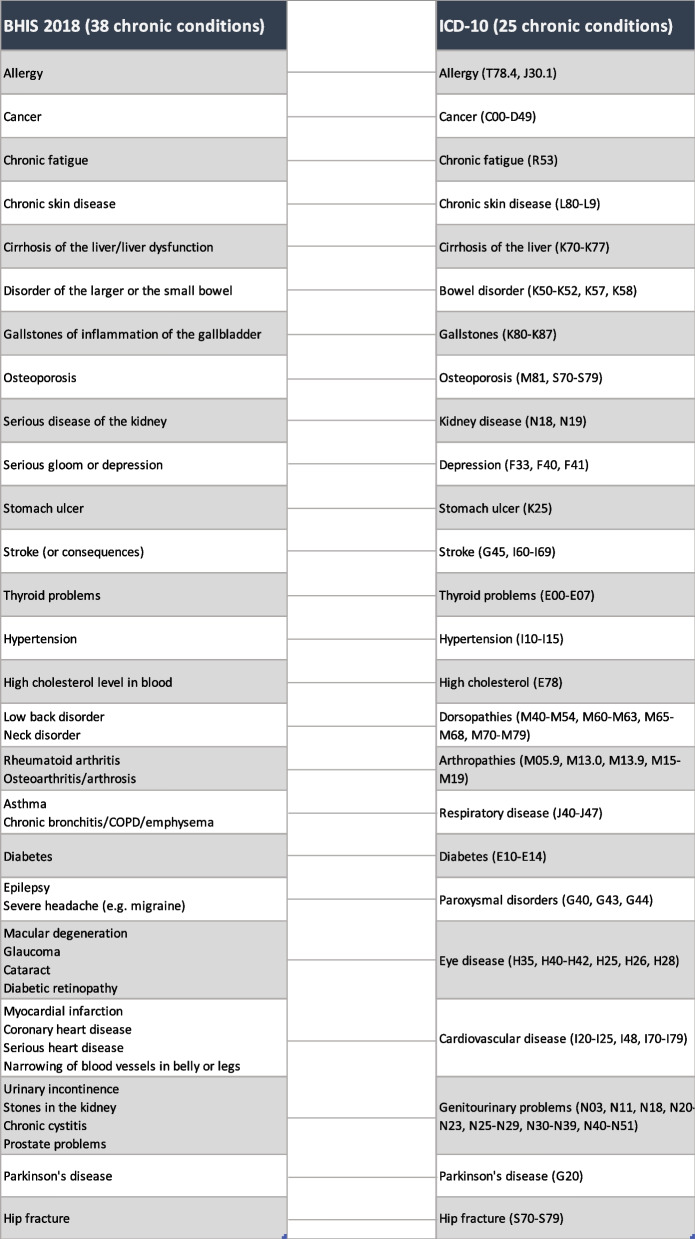
Classification of chronic conditions

### Multimorbidity measures

We defined multimorbidity as “the coexistence of two or more (groups of) chronic conditions” based on our refined list of conditions [3]. To determine multimorbidity, we summed the total number of (groups of) conditions of each respondent.

To report the specific types of multimorbidity combinations, we formed all possible dyads and triads from the list of 25 chronic conditions. However, after assessing the prevalence of single chronic conditions, we excluded conditions that had a prevalence rate of 1% or lower; which were gallstones, cirrhosis of the liver, kidney disease, stroke, hip fracture and Parkinson’s disease. We focused on the more prevalent single chronic conditions, which subsequently formed the more prevalent dyads and triads to enhance the robustness of the results. With fewer observations, statistical fluctuations and random variations can have a more significant impact on the results. This can lead to increased uncertainty and a higher likelihood of producing results that are not representative of the true population estimates. Moreover, the prevalent morbidity combinations contribute more to the burden of disease. In all, we included 171 dyads and 969 triads formed by the list of 19 chronic conditions. The included single chronic conditions, dyads and triads are listed in Additional file 1.

### Timeframe

Each participant’s self-reported health status applies to the 12-month timeframe prior to the date of the interview. In this study, we included the costs of all participants from 2017 to 2020.

### Cost measures

A health system costing perspective was applied in this study. Total cost of healthcare as recorded in the IMA database included the amount reimbursed by insurance and co-payments/supplements paid by the patient. The extracted costs represent all-cause direct healthcare costs on a per-patient basis, but the precise health reason for which medical acts were performed was not directly retrievable from the data.

The included costs were mainly direct medical costs. To a smaller extent, direct non-medical costs were also included, more specifically those which were partially reimbursed and for which information was available in the IMA database. The estimation and reporting of the indirect cost of multimorbidity were conducted in a distinct study, as the data source for addressing this research question differed from the ones utilised in the current study.

Direct costs included direct medical cost (PROCEDURE_GROUP code 1–31, 35–41, 43–83, 85) and direct non-medical cost (code 32–34, 42, 84). The list of value labels is presented in Additional file 2.

Direct medical costs may include:

o Outpatient (medical acts by GPs, specialists and outpatient clinics, physiotherapy, occupational therapy, speech therapy, rehabilitation clinics, etc.)
o Inpatient (stays in general hospitals, specialised psychiatric and neurological hospitals (including day-patient treatment), procedures taking place in the hospital such as dialysis, surgery, etc.)
o Emergency care (intensive care unit (ICU), ambulance, oxygen, etc.)
o Pharmacy (drugs)
o Others (equipment, etc.)

Direct non-medical cost may include:

o Food supplement
o Parking fees

### Data preparation

We summed all-cause healthcare costs by individual per year. The IMA database exclusively contained data concerning variable costs associated with hospital care, i.e. costs related to the specific interventions conducted during the hospitalisation. The IMA dataset did not incorporate hospital fixed cost, which is the fixed amount that covers the cost of the stay (e.g., infrastructure) and care in the hospital. The amount of fixed cost depends on the type of hospital, the services received and the number of nights/days hospitalised. This amount is usually paid out directly to hospitals by health insurance funds, therefore hospital fixed costs were additionally added on for individuals that were hospitalised during each year. To estimate the fixed portion, the number of hospitalisations per patient per year was multiplied by the publicly available average annual 100% per diem cost according to the type of hospitalisation [25, 26]. The resulting fixed costs were then added to the variable hospital costs to obtain the total hospitalisation costs used in this analysis. To enable comparison, all costs from 2017 to 2020 were inflated to 2022 Euro using the Consumer Price Index [27].

### Statistical analyses

Unadjusted subgroup differences were assessed using *t*-tests. Generalised linear models with quasi-poisson distribution and log link function were constructed to estimate the effects of dyads/triads on healthcare costs. Statistical analyses were performed in R through RStudio (2023.03.0) [28]. The complex survey design schemes of the BHIS and sampling weights were accounted for in our survey design object to ensure representativeness of data on the population level. Confidence intervals were calculated using the profile likelihood method [29].

To contain the number of independent parameters, only chronic conditions with a prevalence rate greater than 2.5% were included. Interaction terms are additional variables created by multiplying two or more predictor variables together – in this case they are variables that signify the chronic conditions. Interaction terms were introduced for the most prevalent dyads and triads. Prevalence cut-offs of 2.0% and 1.5% were used, respectively, to ensure parameter identifiability and sufficient observations. The prevalence cut-offs followed a progressive pattern for triads, dyads and single conditions, reflecting the progressively changing prevalence of participants with three, two and one chronic conditions.

Variable selection was facilitated by the filter method which mitigates the risk of overfitting, while striking a balance between including major predictors of interest, maintaining accuracy and ensuring computational efficiency [30]. Filter methods first assess the significance of predictors independently of the predictive models and then proceed to build models using only the predictors that met a certain criterion [31]. The relationship between each predictor and the outcome was determined through the receiver operating characteristics (ROC) curves. If a linear model was fitted, then the absolute value of the *t*-value for the predictor’s slope was examined [32, 33]. Otherwise, a loess smoother was fitted to the predictor and the resulting *R*^2^ statistic was calculated to determine variable importance relative to the intercept-only null model [33].

We constructed three mixed models to separately assess the effects of single chronic conditions, dyads and triads on healthcare costs. The model for single conditions consisted of 19 chronic conditions and six important determinants of healthcare costs; including age, sex, highest education level in the household, financial burden of medical treatment (i.e., a heavy burden/somewhat a burden/not a burden at all/no one in the household needed medical examinations or treatments), number of comorbidities and year. The dyad model replicated the single condition model structure while incorporating 58 additional dyad interaction terms. Similarly, the triad model mirrored the dyad model and included 41 triad interaction terms. A list of the included chronic conditions, dyad and triad interaction terms is supplied in Additional file 1. Model outputs are presented in Additional file 3.

The mixed models for single chronic conditions, dyads and triads were constructed based on these equations, respectively:

$${log (cost) }_{ij} = {\beta }_{{0}_{single}} + {\sum }_{m=1}^{M}{\beta }_{ijm}{chronic \,diseases}_{ijm} + {\sum }_{q=1}^{Q}{\beta }_{ijq}{covariates}_{ijq} + ({u}_{ij}+ {\varepsilon }_{i})$$

and:

$${log (cost) }_{ij} = {\beta }_{{0}_{dyads}} + {\sum }_{m=1}^{M}{\beta }_{ijm}{chronic\, diseases}_{ijm} + {\sum }_{n=1}^{N}{\beta }_{ijn}{dyad\, interactions}_{ijn} + {\sum }_{q=1}^{Q}{\beta }_{ijq}{covariates}_{ijq} \hspace{0.17em}+\hspace{0.17em}({u}_{ij}+ {\varepsilon }_{i})$$

and:

$${log (cost) }_{ij} = {\beta }_{{0}_{triads}} \hspace{0.17em}+\hspace{0.17em}{\sum }_{m=1}^{M}{\beta }_{ijm}{chronic\, diseases}_{ijm}+ {\sum }_{n=1}^{N}{\beta }_{ijn}{dyad\, interactions}_{ijn} + {\sum }_{p=1}^{P}{\beta }_{ijp}{triad\, interactions}_{ijp} + {\sum }_{q=1}^{Q}{\beta }_{ijq}{covariates}_{ijq} \hspace{0.17em}+\hspace{0.17em}({u}_{ij}+ {\varepsilon }_{i})$$

Where: ${log (cost) }_{ij}$ denotes the estimated log-transformed value of the cost variable for individual $i$ in household $j$;

${\beta }_{{0}_{single}}$ and ${\beta }_{{0}_{dyads}}$ and ${\beta }_{{0}_{triads}}$ represent the overall fixed effect mean of ${log (cost) }_{ij}$ across all households $j$ for individuals $i$ of the reference category, for single conditions, dyads and triads, respectively; ${chronic\, diseases}_{ijm}$ is a vector representing individual-level chronic conditions and ${\beta }_{ijm}$ are their respective estimated fixed slope coefficients; ${dyad\, interactions}_{ijn}$ is a vector representing all interaction terms of the 58 most prevalent dyads and ${\beta }_{ijn}$ are their respective estimated fixed slope coefficients; ${triad\, interactions}_{ijp}$ is a vector representing all interaction terms of the 41 most prevalent triads and ${\beta }_{ijp}$ are their respective estimated fixed slope coefficients; ${covariates}_{ijq}$ is a vector representing the six individual-level confounding factors of interest and ${\beta }_{ijq}$ are their respective estimated fixed slope coefficients; The random part between brackets contains: ${u}_{ij}$ and ${\varepsilon }_{i}$. The first allows the intercept to vary between households, accounting for the household specific deviations from the overall intercepts ${\beta }_{{0}_{single}}$ or ${\beta }_{{0}_{dyads}}$ or ${\beta }_{{0}_{triads}}$. The second is the idiosyncratic error term, which accounts for the individual deviations in ${log (cost) }_{ij}$ from the household specific intercept.

One of the main goals was to assess the interaction effects (i.e., interaction term coefficients) of coexisting conditions in a dyad/triad and how they influenced healthcare cost. A 95% confidence level was used to assess whether the costs of dyads/triads significantly differed from the combined costs of single conditions/associated dyads, respectively. *P*-values < 0.05 were reported. A significant *P*-value shows that the predicted summed cost is different when conditions coexist compared with when they exist independently. The interaction effect is multidirectional in that each condition simultaneously influences and is influenced by the other condition(s).

In the case of dyads, a negative coefficient signals a sub-additive (antagonistic) interaction, implying that the combined effect of the two conditions was associated with an estimated mean healthcare cost that is lower than the summed cost of the same conditions existing in different individuals. A positive coefficient shows a super-additive (synergistic) interaction, revealing that the combined effect of the two conditions was associated with an estimated mean healthcare cost that is higher than the summed cost of the same conditions existing in different individuals. The expected mean increase is based on the additive effect of each of the conditions individually.

In the case of triads, we interpreted the results slightly differently. Given that a triad comprises three concurrent conditions, it gives rise to three potential combinations of dyads originating from those three conditions. Consequently, a negative coefficient indicates a sub-additive interaction. This suggests that the combined influence of the three conditions is linked to an average healthcare cost estimate that is lesser than the combined costs of the three associated dyads present in separate individuals. Conversely, a positive coefficient signifies the opposite scenario.

### Risk assessment and ethical consideration

The BHIS 2018 was approved by the Ethics Committee of the University Hospital of Ghent on 21 December 2017 (opinion EC UZG 2017/1454). Participation in the BHIS was voluntary, and no formal requirement for written and signed consent was established. Participation itself was considered tantamount to providing consent. This approach aligns with the principles of Good Epidemiological Practice as outlined by the International Epidemiological Association (IEA) guidelines, which stated that formal written consent may be unnecessary when the research takes place in non-threatening settings and where voluntary participation carries no risk of losing potential benefits [34]. Data linkage was authorised by the Information Security Committee (local reference: deliberation no. 17/119 of 19 December 2017, amended on 3 September 2019).

This study was granted ethical approval on 26 July 2021 by the Ethics Committee at the University of Antwerp Hospital (Ethisch Comité UZA/UA), ID 2021–0405.

## Results

### Overview of multimorbidity in Belgium

**Fig. 2:**
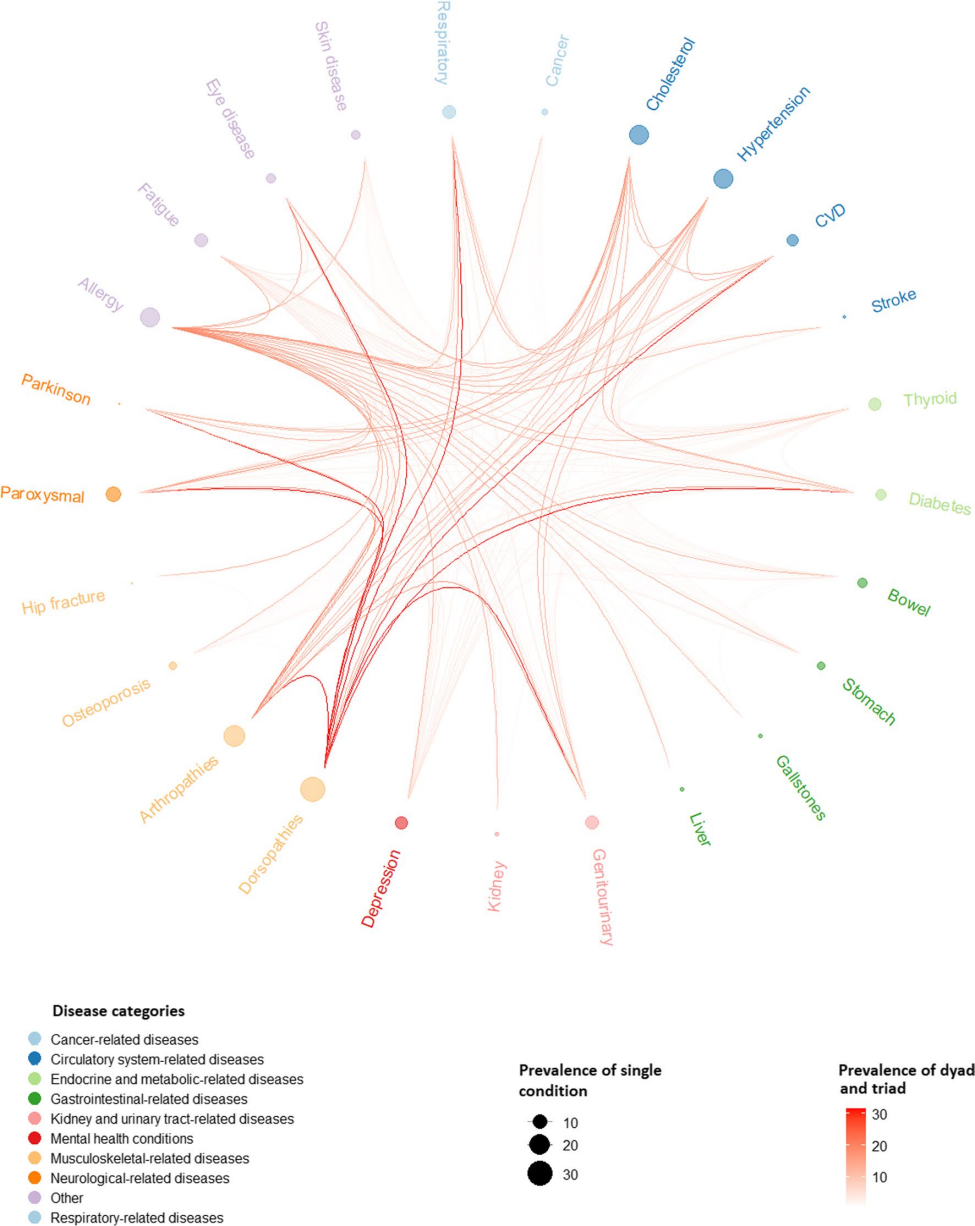
The prevalence and patterns of multimorbidity in 2018. Fatigue: chronic fatigue; respiratory: respiratory disease; cholesterol: high cholesterol level; CVD: cardiovascular disease; thyroid: thyroid problems; bowel:  bowel disorder; stomach: stomach ulcer; liver: cirrhosis of the liver; genitourinary: genitourinary problems; kidney: kidney disease; paroxysmal: paroxysmal disorders (i.e., severe headache, epilepsy); Parkinson: Parkinson’s disease

In Fig. 2, the prevalence of individual conditions, dyads and triads is depicted, along with the patterns of dyad and triad combinations. The outer ring of the figure displays dot sizes reflecting the prevalence of individual chronic conditions, while the orange strokes denote dyads and triads, with colour intensity indicating their respective prevalence. The diverse morbidity labels are colour coded based on their respective disease categories. A comprehensive list of all morbidity combinations and their corresponding prevalence rates is presented in Additional file 4.

In 2018, multimorbidity affected approximately 48% (95% CI 46–49) of the population aged 15 and over in Belgium. Circulatory system-related diseases and musculoskeletal-related diseases were the largest morbidity groups. The most frequent single chronic conditions included arthropathies, dorsopathies, hypertension, high cholesterol, and allergy. The most common dyad was arthropathies + dorsopathies, accounting for 14.1% (95% CI 13.1–15.1) of cases. The most prevalent triad was arthropathies + dorsopathies + hypertension, accounting for 5.2% (95% CI 4.6–5.8) of cases.

### Characteristics of the study population

Table 2 presents the characteristics of the study population and the average annual healthcare expenditures in 2022 Euro, per morbidity count. The overall mean age of the study population was 49. The mean age increased in subgroups with a higher morbidity count. Among the study population (*N* = 9753), 16% had two chronic conditions, 11% had three chronic conditions and 21% had four or more chronic conditions – with these categories being mutually exclusive. Healthcare expenditure also increased with age (*P* < 0.01) and with morbidity count (*P* < 0.01).

**Table 2:** Characteristics of the study population and average annual healthcare expenditure per morbidity count (in 2022 Euro)

	Overall			0 chronic diseases			1 chronic disease			2 chronic diseases			3 chronic diseases			≥4 chronic diseases		
	%	Mean cost (€)	95%CI	%	Mean cost (€)	95%CI	%	Mean cost (€)	95% CI	**%**	Mean cost (€)	95%CI	%	Mean cost (€)	95% CI	%	Mean cost (€)	95% CI
All	100%	3678	(3460–3897)	31%	1556	(1388–1724)	21%	2438	(2141–2734)	16%	3515	(3093–3937)	11%	4592	(3920–5264)	21%	7807	(7052–8562)
Age, mean (95%CI)	49 (48, 49)			39 (38–40)			46 (45–47)			51 (50–53)			56 (54–58)			62 (60–63)		
15–29 years	21%	1210	(1093–1327)	36%	981	(853–1110)	22%	1034	(820–1249)	14%	1911	(1410–2412)	9%	1863	(1010–2716)	5%	2777	(1939–3615)
30–39 years	15%	1745	(1564–1926)	20%	1450	(1249–1651)	20%	1500	(1158–1842)	14%	2164	(1555–2773)	13%	1930	(1297–2563)	6%	3025	(2057–3993)
40–49 years	16%	2446	(2099–2793)	18%	1258	(1054–1463)	18%	2097	(1605–2590)	18%	2020	(1514–2526)	13%	2346	(1843–2849)	13%	5876	(4178–7573)
50–59 years	17%	3322	(2887–3756)	13%	1160	(1018–1303)	17%	2671	[(2049–3292)	20%	2662	(1890–3434)	20%	4266	(2381–6150)	19%	5907	(4779–7035)
60–69 years	14%	4332	(3765–4899)	8%	2798	(1537–4059)	13%	2943	(2059–3827)	17%	3971	(2992–4950)	19%	4112	(3199–5025)	22%	6239	(4842–7636)
70–79 years	10%	6502	(5763–7241)	4%	4193	(2573–5812)	7%	4599	(3014–6184)	11%	5460	(4109–6811)	15%	6211	(4606–7816)	19%	8579	(7096–10,063)
80+ years	7%	13,068	(11,562–14,574)	2%	6902	(3944–9859)	4%	9459	(6945–11,973)	7%	11,928	(8741–15,114)	11%	11,690	(8742–14,638)	16%	16,147	(13,616–18,679)
Sex																		
Male	48%	3355	(3056–3654)	52%	1316	(1107–1526)	51%	2383	(1937–2829)	47%	3349	(2759–3939)	47%	4662	(3603–5721)	38%	8096	(6833–9359)
Female	53%	3971	(3674–4268)	48%	1815	(1552–2078)	49%	2495	(2114–2875)	53%	3662	(3072–4252)	53%	4531	(3681–5380)	62%	7631	(6691–8571)
Household education																		
No diploma/primary	6%	8786	(7305–10,267)	4%	4991	(2652–7331)	5%	5972	(2849–9096)	6%	6742	(3522–9961)	7%	8058	(5661–10,455)	11%	13,358	(10,259–16,457)
Lower secondary	13%	5061	(4454–5668)	10%	2261	(1803–2718)	12%	3421	(2259–4583)	11%	4865	(3323–6407)	17%	5888	(4360–7416)	18%	8210	(6824–9595)
Higher secondary	33%	3776	(3353–4199)	32%	1667	(1302–2032)	31%	2781	(2238–3324)	36%	3310	(2596–4025)	32%	5238	(3586–6891)	34%	7353	(5998–8708)
Higher	48%	2499	(2287–2712)	54%	1093	(1012–1174)	52%	1725	(1462–1989)	47%	2774	(2371–3176)	44%	3082	(2517–3647)	36%	6160	(5121–7198)
Household income(*)																		
Quintile 1	12%	6974	(5969–7980)	8%	3511	(1912–5109)	8%	5207	(3135–7278)	10%	4706	(3218–6193)	14%	6099	(4134–8063)	20%	10,738	(8689–12,788)
Quintile 2	15%	5432	(4598–6267)	14%	2190	(1625–2755)	12%	3934	(2659–5209)	12%	4241	(2845–5636)	16%	6125	(3824–8426)	19%	9733	(7486–11,980)
Quintile 3	20%	3417	(3036–3799)	18%	1316	(1073–1559)	17%	2560	(1918–3201)	21%	2869	(2157–3582)	20%	5006	(3549–6463)	23%	6071	(5093–7049)
Quintile 4	26%	2795	(2483–3106)	26%	1324	(1068–1580)	26%	1934	(1470–2398)	28%	2946	(2327–3564)	25%	4266	(2560–5973)	23%	5146	(4347–5945)
Quintile 5	29%	1937	(1668–2206)	34%	1025	(924–1127)	37%	1548	(1255–1840)	29%	2286	(1806–2765)	24%	2280	(1721–2838)	14%	5411	(3306–7517)
Region																		
Flanders	57%	3762	(3439–4085)	52%	1545	(1291–1800)	62%	2427	(2001–2853)	62%	3510	(2968–4053)	59%	4370	(3564–5176)	56%	8250	(7077–9424)
Brussels	10%	3436	(3078–3794)	13%	1581	(1375–1787)	9%	3400	(2397–4403)	8%	3296	(2309–4284)	8%	4088	(2987–5190)	8%	7756	(6511–9000)
Wallonia	33%	3604	(3269–3938)	35%	1564	(1281–1847)	29%	2163	(1828–2499)	30%	3584	(2772–4396)	33%	5112	(3691–6533)	36%	7135	(6159–8111)

(*) Quintile 1: < 750; Quintile 2: 751–1000; Quintile 3: 1001–1500; Quintile 4: 1501–2500; Quintile 5: > 2500

The population under study was composed of various age groups, among which individuals aged 70+ made up approximately 17%, and their healthcare cost accounted for around 43% of the total healthcare cost of the studied population. People with multimorbidity constituted nearly half of the studied population and their total healthcare cost constituted 74% of the healthcare cost of the studied population. People aged 70+ with multimorbidity accounted for 27% of the multimorbidity population and around 13% of the general studied population. The healthcare expenses associated with multimorbid individuals aged 70+  contributed to 50% of the total healthcare cost of the multimorbidity population and 37% of the total healthcare cost of the general studied population.

The study population consisted of more females than males (53% *vs* 48%) and more females had multimorbidity than males. On average, female participants incurred higher healthcare expenditure than their male counterparts (*P* = 0.003). However, in the subgroups of people with three or more chronic conditions, the average healthcare expenditure per person was higher for male than female participants (not statistically significant). We assessed the highest level of education attained in the household, as a determinant of health expenditure. Around half of all participants came from households with at least one family member with higher education. Participants from households with a lower level of education incurred higher healthcare costs than those from households with a higher level of education (*P* < 0.001). This was consistent across all morbidity counts. Similar to household education, lower household income quintiles were associated with a higher level of health spending and this was observed across all morbidity counts (*P* < 0.05). Across regions, the overall average healthcare expenditure per person was highest in Flanders, followed closely by Wallonia and Brussels. However, this difference was not statistically significant and there were also no clear trends among morbidity counts.

### The average annual direct costs of single chronic conditions

**Fig. 3:**
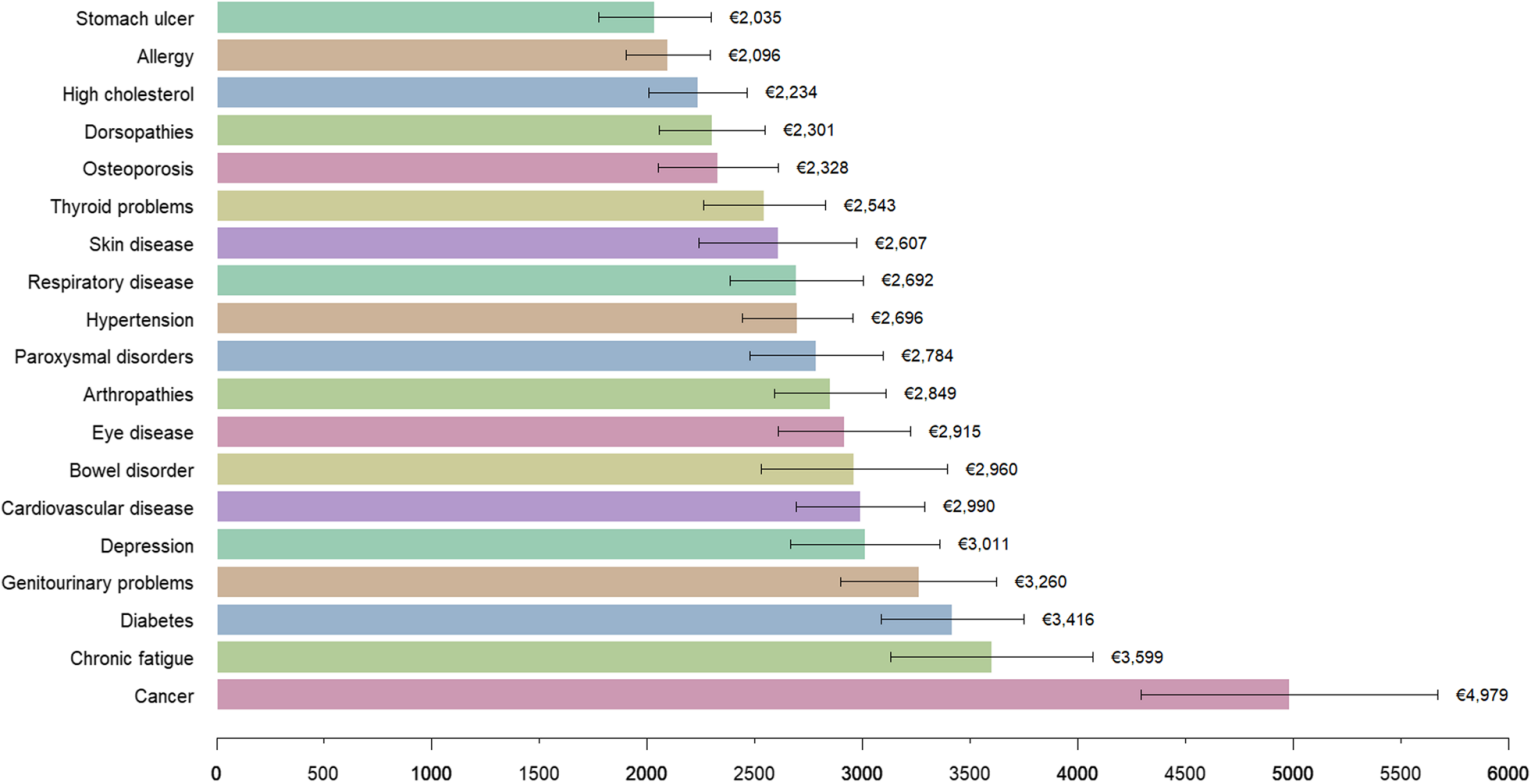
The average annual direct cost per single chronic condition (in 2022 Euro)

The estimated average direct cost per person per year for all single chronic conditions, dyads and triads are presented in Additional file 5. Figure 3 represents the costs associated with the 19 chronic conditions. The colour choices were employed solely for a visually appealing representation and do not serve any classification purpose. The average annual direct cost per person with one chronic condition was €2438 (95% CI 2141–2734). Among the single conditions with the lowest costs, stomach ulcer and allergy stood out, averaging €2035 (95% CI 1524–2546) and €2096 (95% CI 1713–2478) per person per year, respectively. The highest-cost single conditions included cancer, chronic fatigue, diabetes, and genitourinary problems, with average costs of €4979 (95% CI 3629–6329), €3599 (95% CI 2680–4517), €3416 (95% CI 2768–4063), and €3260 (95% CI 2551–3968) per person per year, respectively. As the most prevalent conditions, dorsopathies and arthropathies exhibited average costs of €2301 (95% CI 1818–2783) and €2849 (95% CI 2342–3355) per person per year, respectively.

### The average annual direct costs of dyads

The average yearly cost per person with dyad was €3515 (95% CI 3093–3937).

**Fig. 4:**
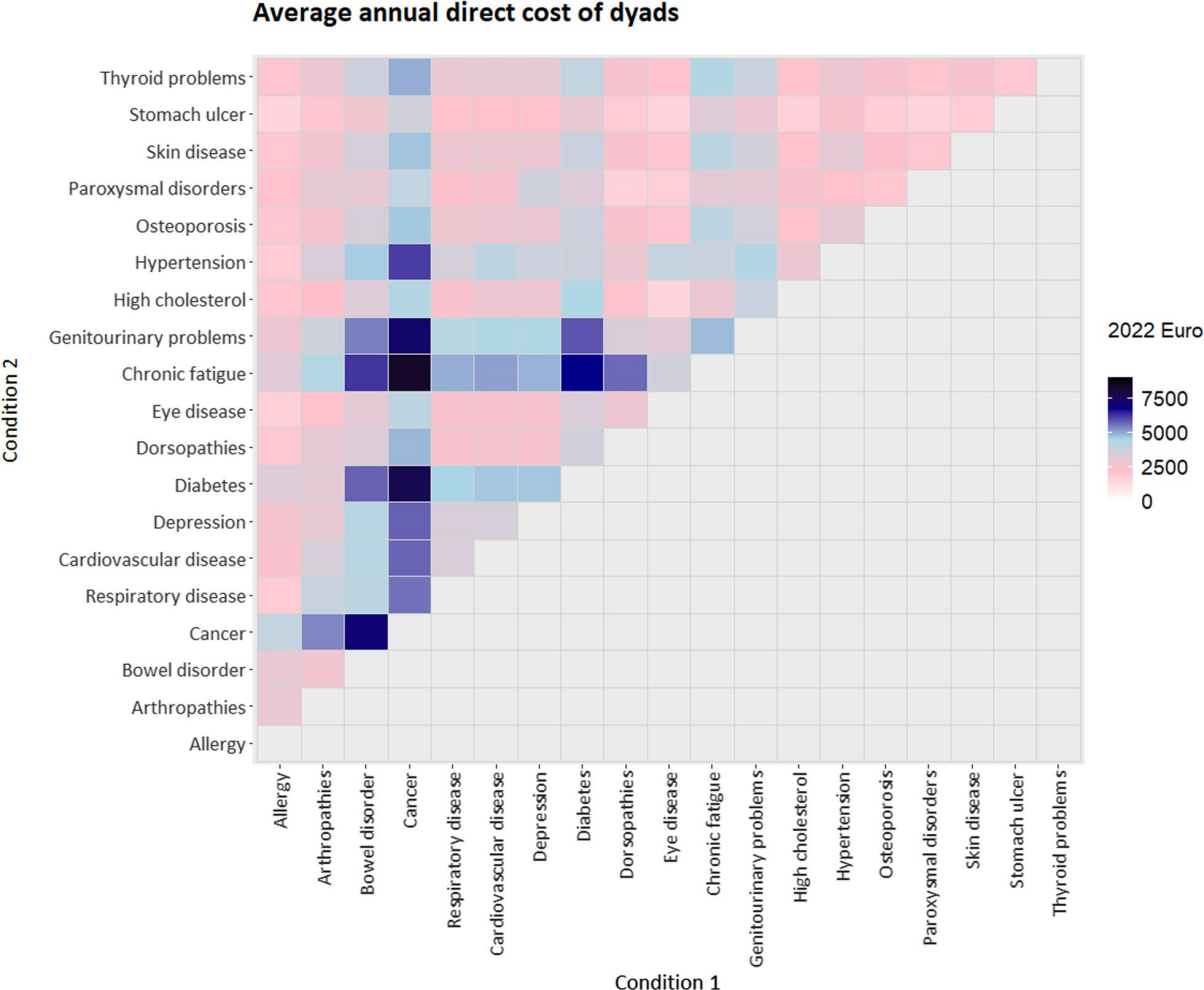
The average annual direct costs of 171 dyads (in 2022 Euro)

The dyads with the highest costs frequently included cancer, diabetes, chronic fatigue, and genitourinary problems (Fig. 4). The top ten most expensive dyads had costs ranging from €5753 (95% CI 3509–7997) for bowel disorder + diabetes to €8345 (95% CI 4998–11,691) for cancer + chronic fatigue.

The low-cost group comprised the ten least expensive dyads – frequently including allergy, stomach ulcer, and osteoporosis – with costs ranging from €1518 (95% CI 912–2124) for eye disease + high cholesterol to €1816 (95% CI 1247–2384) for skin disease + stomach ulcer.

The top ten most prevalent dyads – frequently including arthropathies, dorsopathies, hypertension, and high cholesterol levels – had costs ranging from €1928 (95% CI 1408–2448) for allergy + dorsopathies to €5666 (95% CI 3044–8288) for dorsopathies + chronic fatigue. The most prevalent dyad, arthropathies + dorsopathies, had a cost of €3044 (95% CI 2296–3792). Dorsopathies + paroxysmal disorders was one of the most prevalent and also among the least expensive dyads in terms of direct cost.

### The average annual direct costs of triads

**Fig. 5:**
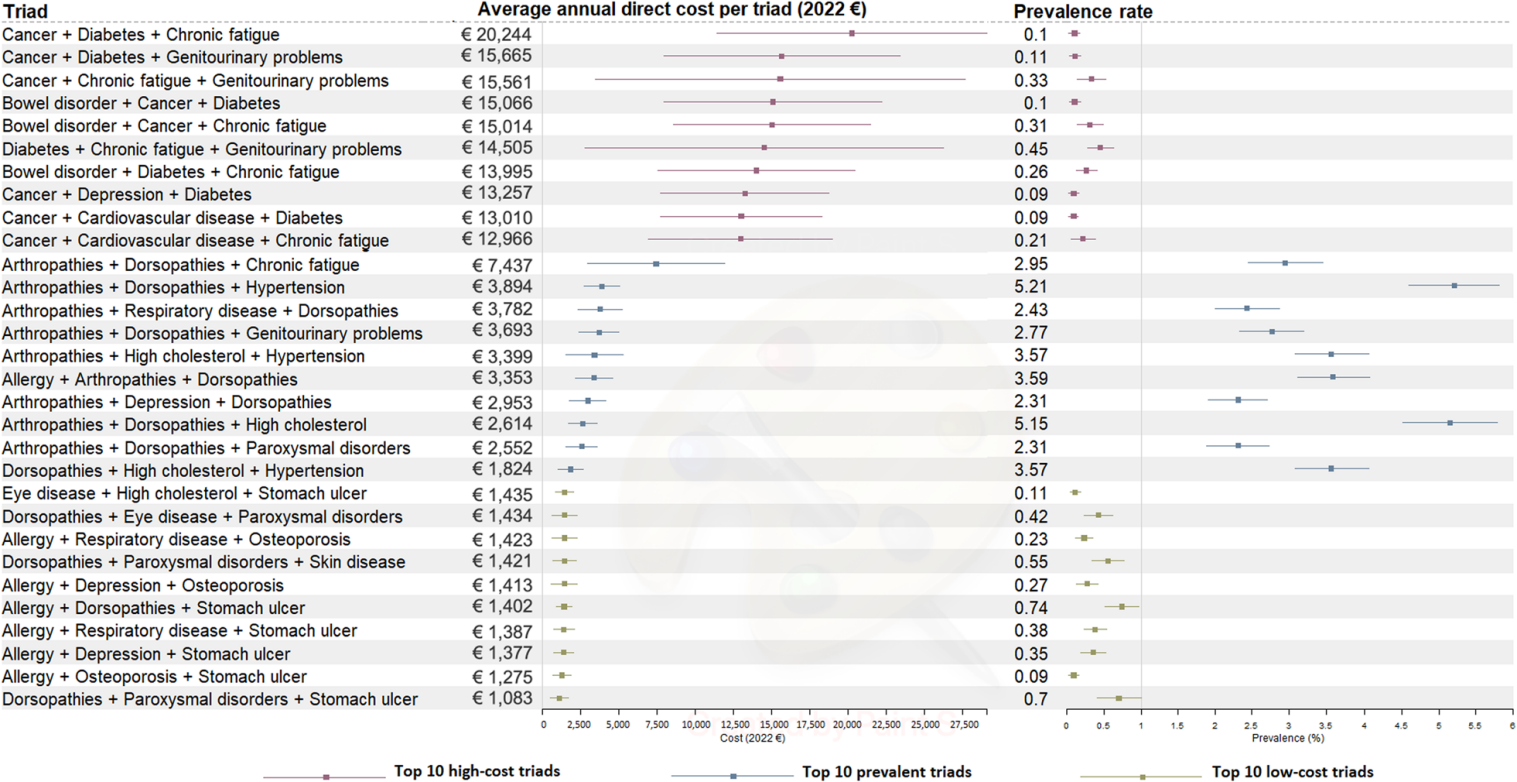
The average annual direct costs of the top high-cost, low-cost and prevalent triads

The average annual cost per person with triad was €4592 (95% CI 3920–5264). Figure 5 shows the expenses associated with the top ten high-cost triads, the top ten low-cost triads and the top ten prevalent triads, along with their respective prevalence rates. Notably, all triads within the top ten high-cost and low-cost categories displayed low prevalence rates, each falling under 1%.

The high-cost group had triads ranging from €12,966 (95% CI 6947–18,985) for cancer + cardiovascular disease + chronic fatigue to €20,244 (95% CI 11,397–29,090) for cancer + diabetes + chronic fatigue.

In contrast, the low-cost group had triads ranging from €1083 (95% CI 503–1663) for dorsopathies + paroxysmal disorders + stomach ulcer to €1435 (95% CI 817–2054) for eye disease + high cholesterol + stomach ulcer.

Triads in the prevalent group had cost ranging from €1824 (95% CI 1010–2638) for dorsopathies + high cholesterol + hypertension to €7437 (95% CI 2966–11,908) for arthropathies + dorsopathies + chronic fatigue, positioning it between the low- and high-cost groups. The most prevalent triad, arthropathies + dorsopathies + hypertension, had a cost of €3894 (95% CI 2725–5063).

### Total cost of multimorbidity on the population level

The dyad with the highest prevalence, arthropathies + dorsopathies, incurred an annual treatment cost exceeding €4 billion, representing 11% of total national health expenditure (Table 3). In contrast, the cost of treating the most expensive dyad, cancer + chronic fatigue, was merely 2%. For triads, the treatment cost of the most prevalent combination, hypertension + arthropathies + dorsopathies, accounted for 5% of national health expenditure. The most expensive triad, cancer + diabetes + chronic fatigue, carried a cost of just 1%.

**Table 3:** Aggregated costs of the most prevalent and expensive dyads and triads (in 2022 Euro)

Type	Morbidity combination	Prevalence rate	Average direct cost per person per year	Total cost on the population level	As % of total health expenditure
Most prevalent dyad	Arthropathies + dorsopathies	14.1% (95% CI 13.1–15.1)	€3044 (95% CI 2296–3792)	€4,070,766,024	11%
Most expensive dyad	Cancer + chronic fatigue	0.8% (95% CI 0.48–1.04)	€8345 (95% CI 4998–11,691)	€633,182,216	2%
Most prevalent triad	Arthropathies + dorsopathies + hypertension	5.2% (95% CI 4.6–5.8)	€3894 (95% CI 2725–5063)	€1,920,488,324	5%
Most expensive triad	Cancer + chronic fatigue + diabetes	0.1% (95% CI 0.02–0.17)	€20,244 (95% CI 11,397–29,090)	€192,003,307	1%

*Total health expenditure was €36,178,000,000 in 2018 [35]

### Interaction effects of dyads and triads on healthcare expenditure

**Fig. 6:**
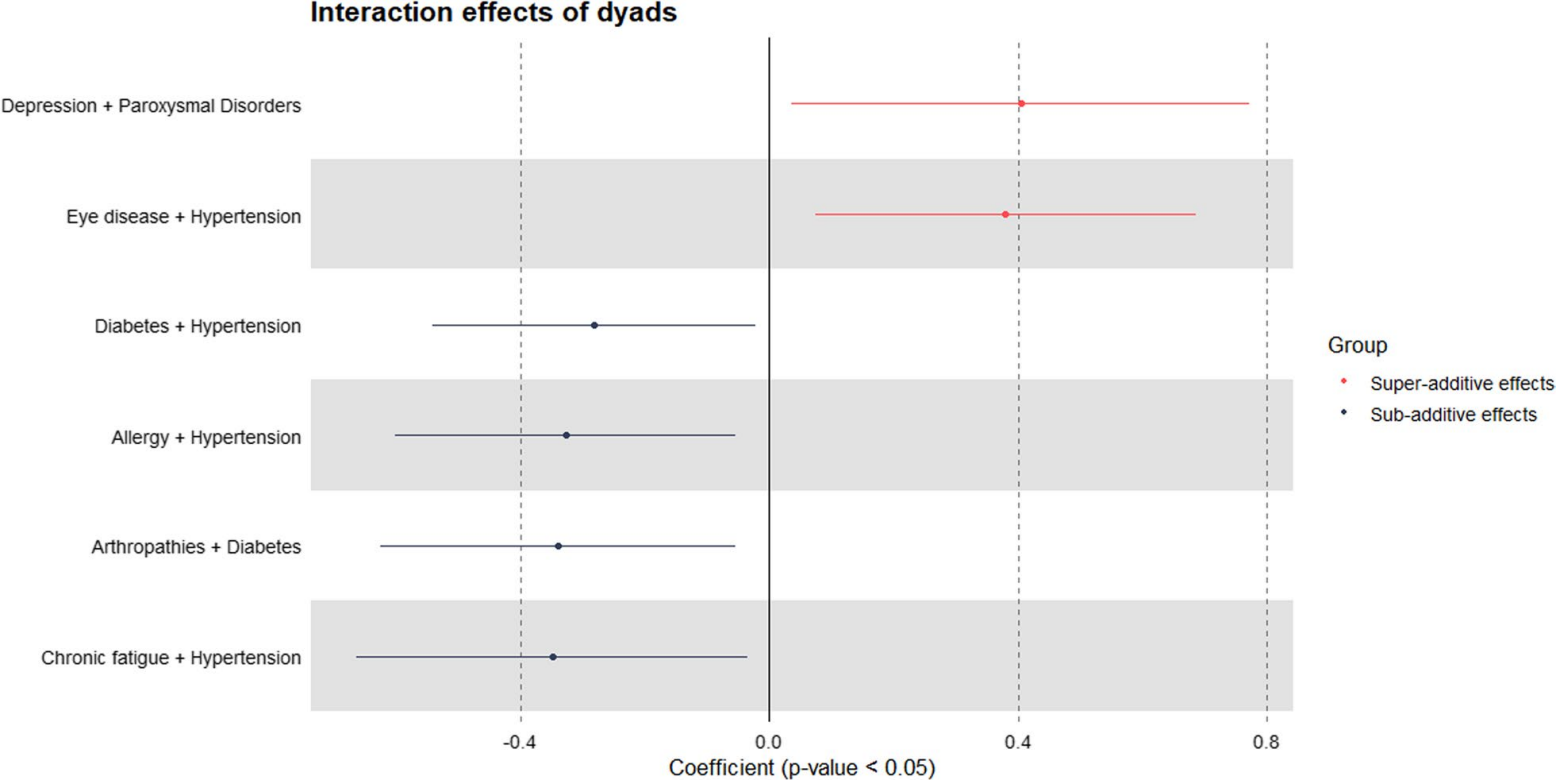
Interaction effects of dyads on healthcare expenditure

Of the 58 most prevalent dyads, six dyads showed significant interaction. Figure 6 shows the coefficients of these six dyads on the log-scale. Among dyads with significant effects, most produced a sub-additive effect, indicating a reduced overall average cost compared with single conditions. The four dyads with sub-additive interaction effects were diabetes + hypertension, allergy + hypertension, arthropathies + diabetes, and chronic fatigue + hypertension. The two dyads with super-additive interaction effects were depression + paroxysmal disorders and eye disease + hypertension.

**Fig. 7:**
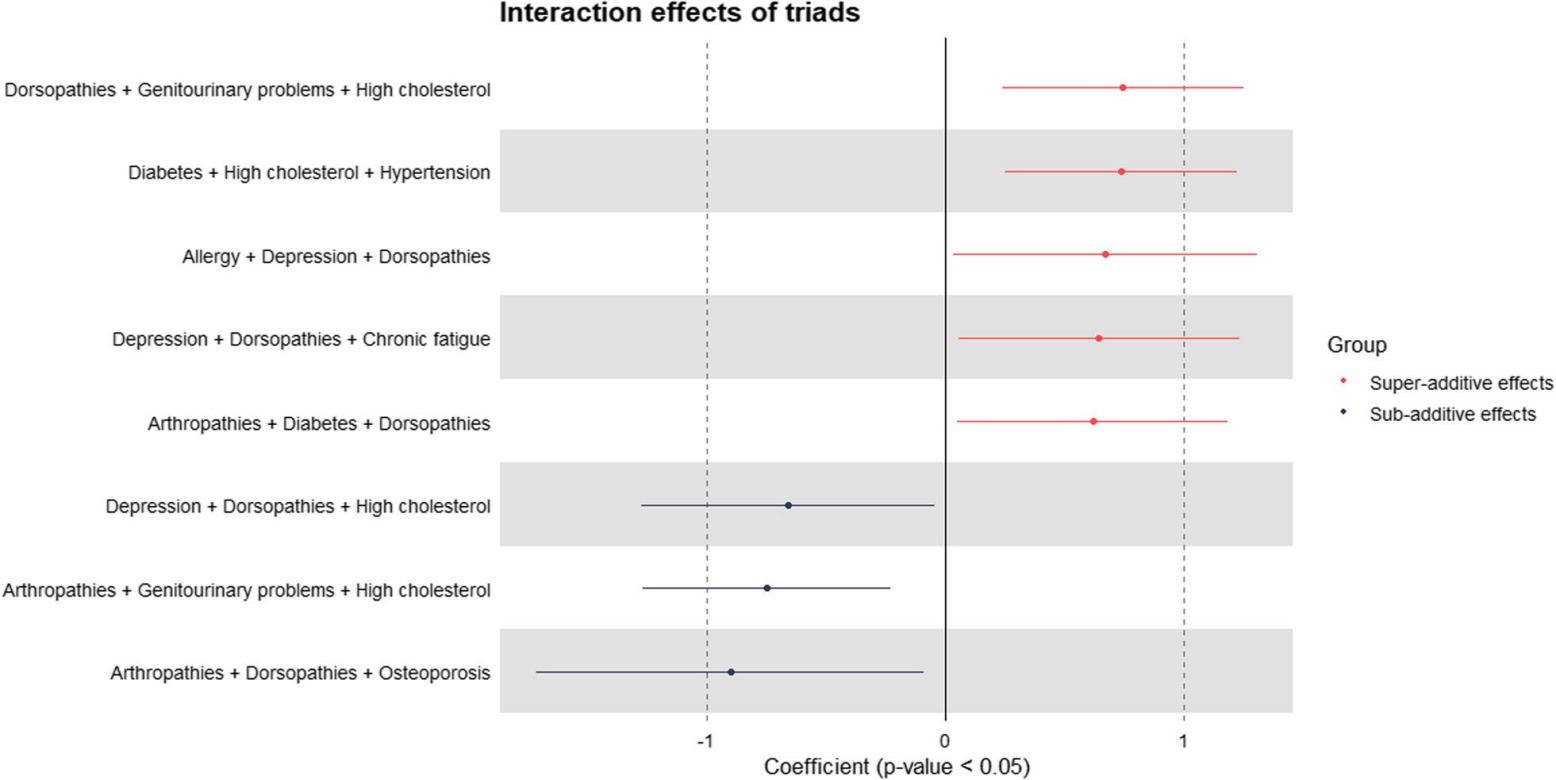
Interaction effects of triads on healthcare expenditure

Of the 41 most prevalent triads, eight showed significant interactions (Fig. 7). Contrary to the interaction patterns seen in dyads; in triads, most had super-additive effects (five triads). Sub-additive effects were found in three triads. The top three triads with super-additive interaction effects were dorsopathies + genitourinary problems + high cholesterol, diabetes + high cholesterol + hypertension, and allergy + depression + dorsopathies. The three triads with sub-additive interaction effects were arthropathies + dorsopathies + osteoporosis, arthropathies + genitourinary problems + high cholesterol, and depression + dorsopathies + high cholesterol.

Overall, with these model specifications and among prevalent morbidity combinations, many of the multimorbidity interactions were insignificant, indicating that the predicted cost of a patient with multimorbidity did not differ significantly from the summed cost of the same conditions existing in different patients.

## Discussion

### Summary of findings

This study provides insights into numerous multimorbidity profiles in Belgium. In addition to examining the cost by morbidity counts as well as demographic and socioeconomic subgroups, the study explored the costs of a large number of dyads (171) and triads (969) associated with the 19 most prevalent chronic conditions. People with multimorbidity constituted nearly half of the studied population and their total healthcare cost constituted around three quarters of the healthcare cost of the studied population. The cost of multimorbidity increased with age and morbidity count, and individuals with lower socioeconomic status were more prone to higher healthcare costs than those of higher socioeconomic status. The most common dyad, arthropathies + dorsopathies, with a prevalence rate of 14%, accounted for 11% of the total national health expenditure. The most frequent triad, arthropathies + dorsopathies + hypertension, with prevalence rate 5%, contributed 5%. Prevalent morbidity combinations, rather than high-cost ones, made a greater contribution to total national health expenditure. The average annual direct cost per person with dyad was €3515 (95% CI 3093–3937), while the average annual direct cost per person with triad was €4592 (95% CI 3920–5264). Dyads and triads associated with cancer, diabetes, chronic fatigue, and genitourinary problems had the highest costs. In most cases, the cost associated with an individual with multimorbidity was lower or not substantially different from the combined cost of the same conditions observed in separate patients.

### General trend of multimorbidity and cost

In 2018, multimorbidity affected approximately 48% of the population aged 15+ in Belgium. This rate surpassed the reported global multimorbidity prevalence of 37.2% (95% CI 34.9–39.4) in the community setting, as well as the regional rate for Europe of 39.2% (95% CI 33.2–45.2) [36]. However, it is important to approach these comparative statements with caution. The presence of heterogeneity in study methodologies, sample selections, data collection approaches, definitions of multimorbidity and the scope of included chronic conditions introduce challenges in directly comparing prevalence rates of multimorbidity.

The five most common chronic conditions in dyads and triads were arthropathies, dorsopathies, hypertension, high cholesterol, and allergy. Several results are consistent with those from the United States, England and France [37–39]. In the United States, arthritis, high cholesterol, and hypertension were also the most common in multimorbidity, alongside diabetes [37, 38]. Hypertension was reported as the most common condition in morbidity combinations in England, alongside diabetes, chronic kidney disease, and asthma [17]. However, chronic kidney disease had low prevalence in Belgium. Diabetes and asthma (as part of the respiratory disease group) were also common, but secondary to those mentioned.

This study supports previous evidence indicating that the cost of multimorbidity increases with age and morbidity count [40–45]. However, after adjusting for morbidity counts, adding age to the analysis did not significantly improve the ability to explain the variation in costs [46]. Nevertheless, individuals aged 70+ who were affected by multimorbidity played a significant role in terms of healthcare costs. Despite constituting a smaller portion of the total population, this group accounted for a substantial share of healthcare expenditures. This underscores the importance of addressing the unique healthcare needs and challenges faced by the elderly population, particularly those dealing with multiple health conditions simultaneously.

Further, our study confirms that multimorbid individuals of lower socioeconomic status had higher healthcare cost compared with those of higher status. Similar research in the United Kingdom also showed that healthcare cost increased with greater levels of deprivation; conversely, individuals from higher socioeconomic backgrounds were more likely to experience better health, leading to lower care needs [47–49].

### Cost of multimorbidity in Belgium in comparison with other countries

In comparison with the few high-income countries for which data are available, the average cost of multimorbidity per person in Belgium appears to be lower, with dyads and triads costing an average of €3515 and €4592 per person per year, respectively. In England, the average annual cost per person with dyad was €5013 (£3717) and with triad was €7116 (£5276), but this only included secondary care costs and excluded primary care and pharmaceutical expenses – the actual cost may be much higher [17]. In the United States, the median costs of dyads and triads were €6751 ($6208) and €8892 ($8177), respectively [37, 50–52]. The most expensive dyad in our study was cancer + chronic fatigue, with an estimated cost of €8345; which was lower than the estimated €11,381 for cancer + neurological diseases in New Zealand [53]. The most prevalent dyad in our study, arthropathies + dorsopathies, cost €3044 – lower than the cost of treating osteoarthritis + back pain in Sweden (€5358) [54]. One of the most prevalent triads in our study was dorsopathies + high cholesterol + hypertension (€1824), the cost of which was significantly lower than the estimated cost of treating low back pain + hypertension + hyperlipidemia (€22,906) in the United States [55]. Comparing our findings with those of other studies is challenging because few other studies have a comparable scope. Further, it should be noted that comparing costs between countries is difficult due to variations in the disease burden, methodology, data collection, sample representativeness and differences in healthcare systems [2]. Despite this, on average, the cost of multimorbidity in Belgium was found to be notably lower than that of other countries with similar economic contexts.

In our study, the dyads associated with the highest costs predominantly consisted of cancer, diabetes, chronic fatigue, and genitourinary problems. These results are, in part, consistent with previous studies that have identified cancer and diabetes, as standalone conditions, associated with high costs, and dyads/triads that included these conditions were also costly to treat [2, 53, 56–58]. However, the reasons why chronic fatigue and genitourinary problems frequently appeared in the top most expensive dyads/triads are less obvious. Further investigation into literature found that chronic fatigue is a complex chronic illness that causes widespread pain, cognitive impairment, can incapacitate individuals for a long period of time, and with a poor prognosis [59]. Diagnosis relies on assessing patient-reported symptoms and extensive testing to exclude other illnesses or factors, as there is no specific laboratory-based diagnostic test [59]. Extensive testing and the impact on quality of life leading to possible homecare service use, to some extent, explain the high cost of chronic fatigue. Genitourinary problems encompass a broad range of conditions, including urinary incontinence, kidney stones, chronic cystitis and prostate problems. Studies have indicated that patients with genitourinary problems not only incurred high healthcare cost for testing and treatment, but also for behavioural therapy, devices and routine care items [60, 61].

In the low-cost group, common chronic conditions within the combinations were allergy, stomach ulcer, and osteoporosis. Allergy is often excluded from multimorbidity studies, making it difficult to compare the cost of combinations involving allergy across countries. As the health outcomes used in this study was self-reported by patients, it was uncertain whether the person received a clinical diagnosis and whether healthcare was sought. Furthermore, for many types of allergies, avoidance of the suspected allergen is the only treatment, and healthcare is typically only sought in the event of a reaction [62, 63]. This may explain the relatively low healthcare cost associated with allergy. Regarding stomach ulcers, complications are uncommon and most cases are treated with pharmacotherapeutics [64, 65]; thus it is reasonable that healthcare costs are relatively low. Regarding osteoporosis, a study conducted in Belgium in 2004 reported having osteoporosis cost €535 per person per year and the author recognised that this figure is low, possibly because osteoporosis remains under-treated in Belgium [66]. Hence, factors such as underreporting, limited healthcare utilisation and cost-effective management contribute partially to the lower costs observed in certain dyads and triads.

### The importance of interaction effects in multimorbidity costing

To ensure precise estimation in multimorbidity costing studies, it is essential to consider interaction effects to avoid the potential risks of overestimating or underestimating cost. Few studies have explored the interaction of conditions and its effect on cost [15, 39, 67]. In our study, the cost of most dyads and triads did not differ significantly from the summed cost of the same conditions existing in different individuals. For those that differed significantly, more sub-additive effects were observed in dyads and super-additive effects in triads. For dyads, one out of two pairs with super-additive interaction effects was discordant and one out of four with sub-additive effects was concordant. For triads, four out of five triads with super-additive interaction effects were discordant and one out of three with sub-additive effects was concordant. Concordant conditions share similar pathophysiologic risks or disease management plans, while discordant conditions are those with unrelated/indirectly related pathophysiologic risks and disease management plans [68, 69]. A third type – dominant conditions – are severe conditions that may limit life expectancy or require extensive medical treatment [68, 69]. Based on this premise, the majority of our results are reasonable as they align with this classification. For example, having eye disease + hypertension (discordant) or dorsopathies + genitourinary problems + high cholesterol (discordant) increased spending, while having diabetes + hypertension (concordant) or arthropathies + dorsopathies + osteoporosis (concordant) reduced spending. Although this does not enable us to explain all of our findings, it serves as a foundation for investigating the healthcare-seeking behaviour of individuals with dyads or triads that displayed super- or sub-additive healthcare expenditure. More frequent utilisation, complex disease trajectories or complications, polypharmacy and inadequate coordination between services are potential explanations why some dyads/triads resulted in super-additive healthcare spending [7, 70, 71].

However, our study showed that most dyads and triads resulted in lower or comparable healthcare costs to the summed cost of the same conditions in different individuals. Although this could be perceived as positive news from an economic perspective, it could also indicate that patients with multimorbidity are “backgrounding” one condition for another and may not be receiving sufficient care for all their conditions. This phenomenon aligns with the Shifting Perspectives Model of Chronic Illness that suggests people living with multimorbidity may place illness in the foreground or the background of their “world”, depending on the context [72].

### Implications for future research, health system and policy

This study assessed morbidity combinations that are most expensive and/or prevalent, which can help to identify where cost savings can be achieved through care reorganisation and prevention. For those with super-additive health expenditure, further research can be conducted into the level of care integration and health seeking behaviour. For concordant diseases, it may be more efficient to seek efficiency gains; for instance, by appointing the concordant disease management to the same medical doctor, resulting in time/cost saving and more effective communication [73]. For common concordant conditions, the first line could be the appropriate level of daily care, with a transmural component of annual visits to a medical specialist [8]. Such models are already in place for single disease care pathways [74]. For those with sub-additive spending, further research can be conducted to understand their health service utilisation patterns. The results can serve as a case study for best practices or identify whether the patient is having unmet needs.

To support decision-makers and researchers to predict and monitor the costs of morbidity combinations, a multimorbidity costing tool can be developed, embedding the models from this study to provide a user-friendly platform that can automatically generate the costs of over a thousand morbidity clusters with fine-tuneable parameters, based on the user’s interest. The potential applications of this tool are extensive, ranging from policy-makers and practitioners to insurance companies, patients and families. Its potential impact in informing health policy and decision-making processes cannot be overstated.

### Strengths and limitations

This study represents the first of its kind in Belgium, at a time when population-level studies on the cost of multimorbidity remain scarce in Europe and globally [2]. By including a large number of morbidity combinations, accounting for both dyads and triads, our study provides a comprehensive assessment of the cost of multimorbidity. While the list of 19 chronic conditions included in the study is not exhaustive, it encompasses the conditions most prevalent in the population, satisfying the criteria of including at least 12 chronic conditions suggested by Fortin et al. for an accurate measurement of multimorbidity [75].

Our use of linked longitudinal data from an exhaustive claim database, a reliable source of information for studying the cost of chronic conditions, increases the reliability of our findings [15, 46, 76]. Notably, linked data is still not widely utilised in research on the cost of multimorbidity, highlighting the novelty of our approach [2]. Furthermore, the use of health insurance claim databases has been endorsed for conducting cost-of-illness studies, further adding to the robustness of our study [77, 78].

Our sample size is relatively large and representative of the population, providing a strong basis for generalisation of findings. We took into account interaction effects, providing more accurate estimations and avoiding the risk of over-/underestimating costs.

There are several limitations and challenges that should be considered when interpreting these findings. Conducting multimorbidity research is inherently challenging due to the vast number of possible morbidity combinations and scenarios. This is further complicated by computational limitations and the need to strike a balance between model fit and parsimony.

The health outcomes were derived from a cross-sectional design survey, which may be limited by patients’ subjective reporting of their health status, unclear diagnoses, overlapping symptoms and other factors that can affect the accuracy of the reported data. For practical reasons, we also assumed that the patients had had the same chronic conditions across all four years, and excluded certain chronic conditions with low prevalence (stroke, cirrhosis of the liver, kidney diseases, Parkinson’s disease, hip fracture, and gallstones) to increase the robustness of our findings. However, this may have led to an overestimation of costs. Regarding covariates, data on proximity to death was insufficient for inclusion, despite its recognised significance as an explanatory factor for healthcare costs, even more so than age [79].

Additionally, diverse methodologies exist for the selection, inclusion and classification of chronic conditions. The list of conditions incorporated in our study was formulated after numerous deliberations within the team, acknowledging that alternative approaches may also exist. For instance, some conditions could be classified as symptoms or risk factors, rather than standalone chronic conditions. While cancer represents a condition characterised by clear and comprehensive clinical manifestations, high cholesterol and hypertension, on the other hand, are risk factors predisposing individuals to the development of future diseases. Chronic fatigue remains a topic of debate within medical circles due to its association with numerous other conditions, presenting varied levels of reporting and diagnostic confirmation. The conditions included in the study exhibit a diverse spectrum in how they manifest clinically, their impact on health outcomes and the diverse physiological pathways underlying each condition. This is inevitable given the complex nature of chronic conditions and multimorbidity, and the lack of a consensus on definitions and terminologies [3, 75, 80]. Moreover, some conditions were collapsed to form broader morbidity groups (Fig. 1) and there may be potential overlaps across groups. The limited number of included conditions could potentially have resulted in an underestimated prevalence of multimorbidity. Nonetheless, our study aimed to capture all “available” conditions from the database, particularly those that could impact a person’s health-related quality of life and incur healthcare expenditure over a long period. Indirect costs, estimated using a different data source, were not presented here but in a separate study.

Finally, although beyond the scope of our study, we find it important to acknowledge the potential impact of COVID-19 on our results. Upon examining our data and consulting official figures from the Ministry of Social Security, we observed only a marginal increase in total healthcare expenditure in 2020 compared with 2019. While COVID-19 may have influenced healthcare expenditure in 2020, our analysis spanned multiple years, suggesting that any effect is likely minimal and largely irrelevant for the purpose of this analysis and the insights it provides. Other minor limitations were the reporting of cost per average year and the assumption that different multimorbidity scenarios were equally affected by any COVID-19 impact over the relatively short time window.

## Conclusions

Prevalent morbidity combinations, rather than high-cost ones, made a greater contribution to total national health expenditure. Our research serves as a starting point for subsequent research on the healthcare-seeking behaviour of individuals with super or sub-additive healthcare expenditure. The models developed in this study can be used to create a user-friendly costing tool for multimorbidity, which can inform health policy and decision-making processes. We draw upon this evidence as a stepping stone to enhance the healthcare system, with an aim to develop more accommodating and cost-effective care for patients with diverse needs. Our study contributes to the scarce literature on this topic in Europe and worldwide.

### Supplementary Information

**Additional file 1. **List of included single chronic conditions and interaction terms for prevalent dyads and triads in the models.

**Additional file 2. **Cost ingredients.

**Additional file 3. **Model outputs.

**Additional file 4. ** Prevalence of singles chronic conditions—dyads—triads.

**Additional file 5. ** Costs of single chronic conditions—dyads—triads.

## Data Availability

The data that support the findings of this study are available from the Intermutualistic Agency but restrictions apply to the availability of these data, which were used under license for the current study, and so are not publicly available. Data are, however, available upon reasonable request and with permission from the Intermutualistic Agency.
