# Supplementary material for: Multimorbidity healthcare expenditure in Belgium: a 4-year analysis (COMORB study)
Source: Health Res Policy Syst. 2024 Mar 22;22:35. doi: 10.1186/s12961-024-01113-x (PMC10960468; doi:10.1186/s12961-024-01113-x)
Supplement: Supplementary file 1 — Additional file 1. List of included single chronic conditions and interaction terms for prevalent dyads and triads in the models. [file 12961_2024_1113_MOESM1_ESM.pdf]

Supplementary File 5 : List of included single chronic conditions, and interaction terms for prevalent dyads and triads in the models

19 single chronic conditions

1. allergy
2. arthropathies
3. bowel disorder
4. cancer
5. cardiovascular disease
6. respiratory disease
7. depression
8. diabetes
9. dorsopathies
10. eye disease
11. chronic fatigue
12. genitourinary problems
13. high cholesterol level
14. hypertension
15. osteoporosis
16. paroxysmal disorders
17. chronic skin disease
18. stomach ulcer
19. thyroid problems

58 interaction terms for prevalent dyads

1. dorsopathies*arthropathies
2. high cholesterol level*dorsopathies
3. allergy*dorsopathies
4. hypertension*high cholesterol level
5. hypertension*dorsopathies
6. hypertension*arthropathies
7. high cholesterol level*arthropathies
8. dorsopathies*paroxysmal disorders

9. chronic fatigue*dorsopathies
10. allergy*arthropathies
11. dorsopathies*genitourinary problems
12. depression*dorsopathies
13. dorsopathies*respiratory disease
14. arthropathies*genitourinary problems
15. allergy*respiratory disease
16. allergy*high cholesterol level
17. chronic fatigue*arthropathies
18. allergy*paroxysmal disorders
19. allergy*hypertension
20. hypertension*genitourinary problems
21. arthropathies*respiratory disease
22. thyroid*dorsopathies
23. high cholesterol level*genitourinary problems
24. high cholesterol level*diabetes
25. chronic fatigue*depression
26. arthropathies*paroxysmal disorders
27. arthropathies*cardiovascular disease
28. dorsopathies*cardiovascular disease
29. hypertension*diabetes
30. high cholesterol level*cardiovascular disease
31. thyroid*arthropathies
32. bowel disorder*dorsopathies
33. chronic fatigue*paroxysmal disorders
34. depression*arthropathies
35. hypertension*cardiovascular disease
36. allergy*chronic fatigue
37. arthropathies*eye disease
38. dorsopathies*eye disease
39. chronic fatigue*high cholesterol level
40. high cholesterol level*respiratory disease

41. osteoporosis*arthropathies
42. dorsopathies*diabetes
43. hypertension*respiratory disease
44. arthropathies*diabetes
45. high cholesterol level*paroxysmal disorders
46. allergy*genitourinary problems
47. chronic fatigue*hypertension
48. chronic fatigue*genitourinary problems
49. osteoporosis*dorsopathies
50. depression*paroxysmal disorders
51. allergy*depression
52. depression*high cholesterol level
53. hypertension*eye disease
54. thyroid*high cholesterol level
55. thyroid*hypertension
56. hypertension*paroxysmal disorders
57. bowel disorder*arthropathies
58. high cholesterol level*eye disease

41 interaction terms for prevalent triads

1. hypertension*dorsopathies*arthropathies
2. high cholesterol level*dorsopathies*arthropathies
3. allergy*dorsopathies*arthropathies
4. hypertension*high cholesterol level*arthropathies
5. hypertension*high cholesterol level*dorsopathies
6. chronic fatigue*dorsopathies*arthropathies
7. dorsopathies*arthropathies*genitourinary problems
8. dorsopathies*arthropathies*respiratory disease
9. depression*dorsopathies*arthropathies
10. dorsopathies*arthropathies*paroxysmal disorders
11. allergy*high cholesterol level*dorsopathies
12. dorsopathies*arthropathies*cardiovascular disease
13. chronic fatigue*depression*dorsopathies

14. chronic fatigue*dorsopathies*paroxysmal disorders
15. thyroid*dorsopathies*arthropathies
16. allergy*dorsopathies*paroxysmal disorders
17. hypertension*dorsopathies*genitourinary problems
18. hypertension*high cholesterol level*diabetes
19. allergy*hypertension*dorsopathies
20. allergy*dorsopathies*respiratory disease
21. osteoporosis*dorsopathies*arthropathies
22. high cholesterol level*dorsopathies*genitourinary problems
23. allergy*high cholesterol level*arthropathies
24. hypertension*arthropathies*genitourinary problems
25. dorsopathies*arthropathies*eye
26. hypertension*high cholesterol level*genitourinary problems
27. allergy*chronic fatigue*dorsopathies
28. chronic fatigue*high cholesterol level*dorsopathies
29. allergy*hypertension*arthropathies
30. chronic fatigue*dorsopathies*genitourinary problems
31. bowel*dorsopathies*arthropathies
32. high cholesterol level*arthropathies*genitourinary problems
33. high cholesterol level*dorsopathies*paroxysmal disorders
34. depression*high cholesterol level*dorsopathies
35. chronic fatigue*hypertension*dorsopathies
36. high cholesterol level*dorsopathies*cardiovascular disease
37. dorsopathies*arthropathies*diabetes
38. hypertension*high cholesterol level*cardiovascular disease
39. allergy*dorsopathies*genitourinary problems
40. hypertension*arthropathies*cardiovascular disease
41. allergy*depression*dorsopathies
