# Supplementary material for: Multimorbidity healthcare expenditure in Belgium: a 4-year analysis (COMORB study)
Source: Health Res Policy Syst. 2024 Mar 22;22:35. doi: 10.1186/s12961-024-01113-x (PMC10960468; doi:10.1186/s12961-024-01113-x)
Supplement: Supplementary file 2 — Additional file 2. Cost ingredients. [file 12961_2024_1113_MOESM2_ESM.pdf]

Procedure group codes

1 Medical fees - Clinical biology
2 Medical fees - Fees / packages
3 Medical fees - Consultations, visits and advice
4 Medical fees - Special services
5 Medical fees - Surgery
6 Medical fees - Gynecology
7 Medical fees - Surveillance
8 Dental fees
9 Fees for nursing practitioners
10 Pharmaceutical specialties - Specialties delivered to non-hospitalized beneficiaries
11 Pharmaceutical specialties - Specialties from the hospital pharmacy, delivered to patients
12 Pharmaceutical specialties - Specialties delivered to beneficiaries hospitalized
13 Pharmaceutical services - Official receipts - Conditional nature
14 Pharmaceutical services - On-call fees
15 Pharmaceutical services - Blood and blood plasma
16 Pharmaceutical services - Medical resources
17 Pharmaceutical services - Medical food
18 Pharmaceutical services - Miscellaneous pharmaceutical services
19 Care by physiotherapists
20 Care by bandagists
21 Care by orthopedists
22 Implants and implantable heart defibrillators
23 Care by opticians
24 Care by audicians
25 Births by midwives
26 Price per maintenance day
27 Military hospital - all-price in
28 Flat rate for maintenance day in the general hospital
29 Dialysis - Doctors
30 Dialysis - Flat rate renal dialysis
31 Dialysis - At home, in a center
32 MRS/MRPA/Day care centers - Rest and care homes
33 MRS/MRPA/Day care centers - Nursing homes for the elderly
34 MRS/MRPA/Day care centers - Day care centers - Coma
35 Mental health care - Psychiatric care homes
36 Mental health care - Sheltered housing initiatives
37 Mental health care - Lump sum for maintenance day in the psychiatric hospital
38 Functional and vocational rehabilitation
39 Special Fund
40 Speech therapy
41 Paediatric medical centres
42 Other accommodation and travel expenses

44 Social maximum invoice
45 Chronic patients
46 Palliative care (patient)
47 Human tissues
48 Adjustments + re-invoicing
49 Palliative care
50 Spa treatments
51 Support for multidisciplinary care, first line
52 Benefits drugs - Oxygen
53 Multidisciplinary teams
54 Smoking cessation
55 Medical centers
56 SP/ALS/Huntington
57 Nursing day prize (sixth state reform)
58 RVT/ROB/Day care centers (excluding sixth state reform)
59 Rehabilitation and retraining (sixth state reform)
60 Multidisciplinary primary care (sixth state reform)
61 Care by bandagists (sixth state reform)
62 Regularizations (regional competence)
63 Psychological care
64 Healthcare innovation
80 Magnetic strips
81 Statistical codes
82 Nomenclature of the framework
83 Article 56 - Dental care for underprivileged children and specific contraceptive intervention for young people
84 Emergency and enteral nutrition co-payments (app. Maximum invoice)
85 Neuropsychiatry ceiling code
86 Wrongly paid amounts
87 Maximum dental rates
88 Wrongly paid amounts charged to ZIV
90 International conventions
